# The Influence of a Plant-Based Diet on Skin Health: Inflammatory Skin Diseases, Skin Healing, and Plant-Based Sources of Micro- and Macro-Nutrients

**DOI:** 10.3390/life14111439

**Published:** 2024-11-07

**Authors:** Mildred Min, Anurag Tarmaster, Apple Bodemer, Raja K. Sivamani

**Affiliations:** 1 Integrative Skin Science and Research, 1491 River Park Drive, Sacramento, CA 95815, USA; 2 College of Medicine, California Northstate University, 9700 W Taron Dr, Elk Grove, CA 95757, USA; 3 Department of Dermatology, University of Wisconsin School of Medicine and Public Health, Madison, WI 53715, USA; 4 Pacific Skin Institute, 1495 River Park Dr Suite 200, Sacramento, CA 95815, USA; 5 Department of Dermatology, University of California-Davis, 3301 C St. #1400, Sacramento, CA 95816, USA; 6 Integrative Research Institute, 4825 J Street, Suite 100, Sacramento, CA 95819, USA

**Keywords:** diet, vegan, plant based, psoriasis, acne, hidradenitis suppurativa, atopic dermatitis, gut–skin axis, gut microbiome

## Abstract

Dietary patterns have been shown to worsen or alleviate several dermatological diseases. A well-balanced, plant-based diet is known to have anti-inflammatory, probiotic, and antioxidant properties, along with weight loss-promoting effects. Moreover, a plant-based diet has a low glycemic load, improving metabolic disease. Due to these qualities, plant-based diets may have beneficial effects on inflammatory skin conditions. In this review, we aim to discuss the possible mechanisms by which a plant-based diet reduces disease severity in psoriasis, acne, hidradenitis suppurativa, and atopic dermatitis. We also aim to clarify how a plant-based diet may influence skin healing and identify sources of vitamins, nutrients, fatty acids, and protein in a well-balanced, plant-based diet. We performed a literature search on PubMed/MEDLINE databases with the following keywords: “plant-based” OR “vegan” OR “vegetarian” OR “meat” OR “diet” AND “psoriasis” OR “hidradenitis suppurativa” OR “acne” OR “atopic dermatitis” OR “skin healing” OR “dermatology”. Our findings demonstrate that plant-based foods may improve inflammatory skin diseases by supporting the gut microbiome, exerting anti-inflammatory effects, providing barrier support, and improving glycemic control. With the proper education, there is an abundance of plant-based food sources or supplements that contain riboflavin, vitamin B12, vitamin A, omega-3 fatty acids, and protein, thereby ameliorating the risk of nutritional deficiencies. Thus, a plant-based diet may have therapeutic potential in dermatology. In spite of the evidence available, there is a paucity of clinical studies focusing specifically on plant-based diets and dermatologic conditions and further investigation is warranted.

## 1. Introduction

Suboptimal nutrition is a known contributor to worsening skin health, as evidenced by dermatological manifestations of nutrient and vitamin deficiencies. For example, vitamin C and essential fatty acid deficiencies have been observed in cases of poor wound healing [1]. Zinc deficiencies have been shown to worsen alopecia and acrodermatitis enteropathica [2]. Riboflavin and niacin maintain cellular function, metabolism, and DNA repair; thus, deficiencies in these vitamins can lead to dermatitis and pellagra, respectively [3]. Moreover, inflammatory skin conditions including psoriasis, acne vulgaris, atopic dermatitis, and hidradenitis suppurativa have been observed to worsen or improve with certain dietary practices [4]. Thus, understanding the role that diet plays in skin health and disease is crucial in order to provide optimal holistic counseling to patients with dermatologic conditions.

One mechanism by which diet influences skin health is via its modulation of the gut microbiome and gut–skin axis. Gut dysbiosis is known to lead to altered immunity and metabolism, therefore worsening skin health and inflammation [5]. For example, in psoriasis and HS, there is a significant association of disease with metabolic syndrome, and emerging evidence suggests that worsened metabolic health correlates with increased disease severity. When metabolic derangements were normalized via diet or weight loss interventions, several studies demonstrated an improvement in disease severity in psoriasis and HS participants [6,7]. Moreover, oral supplementation with probiotics reduced inflammatory markers, decreased Psoriasis Area and Severity Index (PASI) scores, and improved quality of life in psoriatic participants, further supporting the role of the gut microbiome in dermatology [8]. Notably, the literature suggests that plant-based diets may increase beneficial bacteria in the gut, ameliorating gut dysbiosis and promoting overall health [9]. Therefore, plant-based foods may improve skin health through their modulation of gut health.

The consumption of plant-based foods has long been known to be key in maintaining many aspects of overall health, including skin health and function. In one epidemiological study of people aged 70 and over, the increased consumption of fruits and vegetables, olive oil, and nuts and legumes correlated with decreased actinic skin damage [10]. Another Japanese study in women demonstrated an inverse association between rhytids and vegetable intake [11]. Fermented vegetables, such as kimchi, may also act as a probiotic source, contributing to skin health via the gut–skin axis [12]. Plant-based foods are rich in antioxidants, polyphenols, carotenoids, vitamins, and fiber, all of which are key regulators of the gut microbiome and strengthen the skin’s defense against the external environment.

Alternatively, processed meats, refined grains, soft drinks, and alcoholic beverages have been associated with worsening skin wrinkling [10], suggesting a reduced defense against cellular damage and aging. Notably, the consumption of animal-based products has been implicated in inflammatory skin conditions. Dairy products have been thought to be associated with the worsening of acne and HS, likely due to influences on insulin-like growth factor 1 (IGF-1) [13]. In patients with HS and psoriasis, the consumption of red meat has demonstrated worsening of systemic inflammation, driving disease severity [14].

Given this preliminary evidence, a plant-based diet may have therapeutic indications in dermatology and is a vital source of vitamins and minerals necessary for overall health. However, there remains a gap in knowledge of the mechanisms by which a plant-based diet may modulate chronic inflammation and oxidative stress which contribute to disease severity. This review aims to summarize the available evidence for a plant-based diet in dermatology, specifically focusing on its mechanisms in inflammatory skin diseases, skin healing, and plant-based sources of micro- and macro-nutrients.

## 2. Methods

A literature search was performed on the PubMed/MEDLINE databases with the following keywords: “plant-based” OR “vegan” OR “vegetarian” OR “meat” OR “diet” AND “psoriasis” OR “hidradenitis suppurativa” OR “acne” OR “atopic dermatitis” OR “skin healing” OR “dermatology” through July 2024. In July 2024, all studies available in English underwent an initial review. Eligibility criteria for studies included dietary interventions and measures of skin health, based on subjective or objective disease severity. Animal or in vitro studies were included only if they revealed potential mechanisms for plant-based foods that could support future clinical trials. Otherwise, only studies in humans were selected. During the full-text review, additional relevant articles were selected after scanning the citations. Article extraction and analysis were completed by August 2024.

## 3. Results and Discussion

**Figure 1:**
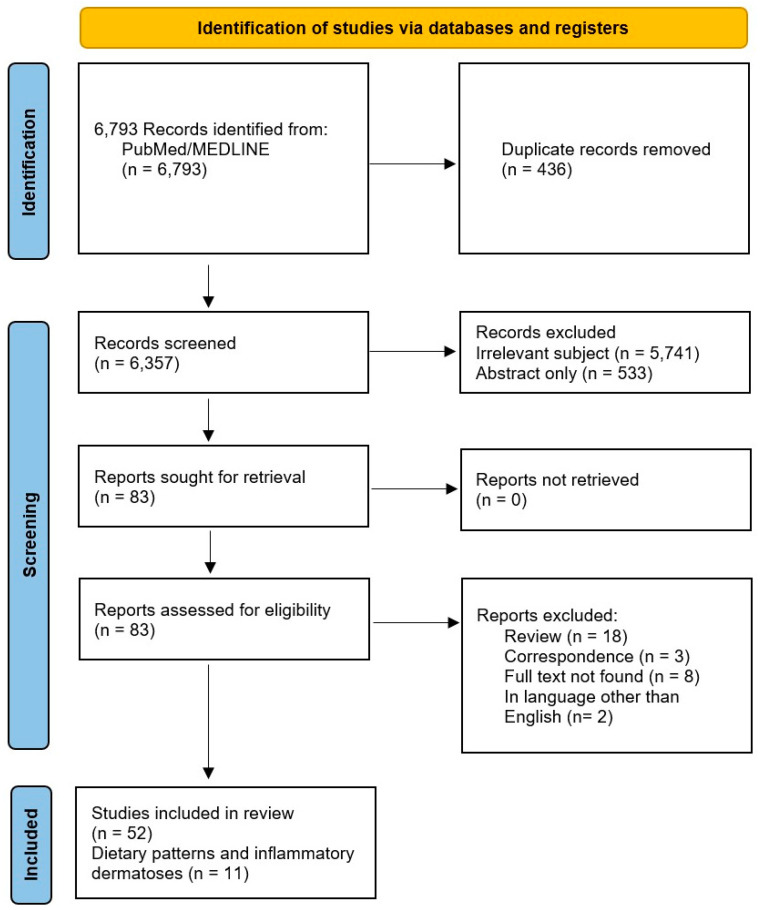
**PRISMA flow diagram of selected studies.** This figure was created in Microsoft Word version 16.90 (Redmond, WA, USA).

A total of 52 studies were extracted for discussion, with eleven studies specifically discussing dietary patterns and inflammatory skin diseases (Figure 1).

**Figure 2:**
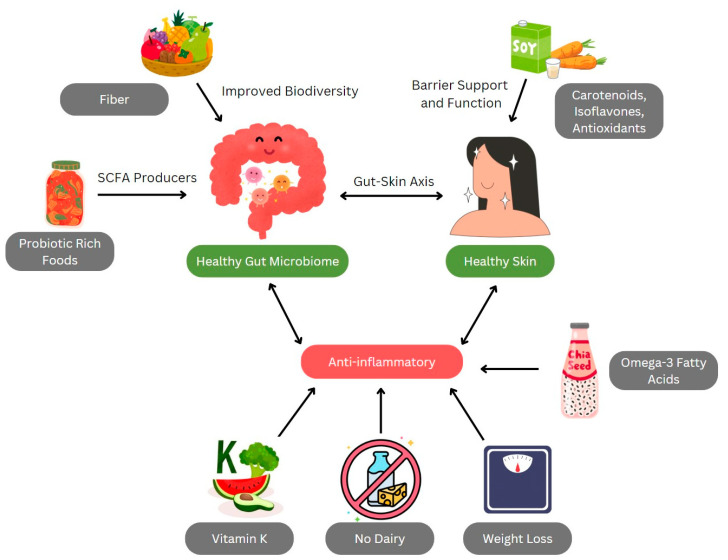
**Proposed mechanisms of a plant-based diet on inflammatory skin diseases.** Plant-based foods are rich in fiber and probiotics which support bacteria that metabolize and produce SCFAs that are key regulators of gut health. Plant-based diets contain vitamin K, eliminate dairy sources, promote weight loss, and improve ratios of omega-3 fatty acids to omega-6 fatty acids, thereby reducing systemic inflammation. Plant-based foods also contain bioactive compounds such as carotenoids, isoflavones, and antioxidants that act on skin barrier support and function. Altogether, these mechanisms lead to a healthier gut microbiome, reduced systemic inflammation, and healthy skin. This figure was created on Canva (Sydney, Australia). Abbreviations: SCFA, short-chain fatty acid.

A summary of the proposed mechanisms for a plant-based diet in inflammatory skin diseases is shown in Figure 2. A summary of the findings for inflammatory skin diseases in this review can be found in Table 1.

### 3.1. Psoriasis

Psoriasis is a chronic inflammatory disease with cutaneous and systemic components. Due to the chronic systemic inflammation driving psoriasis, it is associated with several comorbidities including obesity, metabolic syndrome, kidney disease, and cardiovascular ailments [29]. There is emerging interest in the role of diet and nutrition as a means of managing psoriasis, and this may be partly due to the associated cost and duration of treatment options currently available. Given the increased body mass index (BMI) and adiposity seen in individuals with psoriasis, dietary changes may also improve associated comorbidities, further leading to decreased disease burden.

Several studies have investigated dietary patterns in psoriatic patients to reveal common dietary habits. For example, one cross-sectional study demonstrated that psoriatic patients most commonly follow one of two dietary patterns: (1) mostly processed foods or (2) mostly fresh foods. When comparing the two groups of people with psoriasis, the study found that the second dietary pattern led to a lower waist-to-hip ratio, decreased psoriasis in the skin (measured by Psoriasis Assessment Severity Score (PASS)) [30], and blood pressure within normal limits compared to those who eat mostly processed foods [31]. In a 2016 national U.S. self-reported survey, psoriatic patients reported greater skin improvement after reducing their intake of alcohol, gluten, and nightshade vegetables. Moreover, adding omega-3 fatty acids and oral vitamin D supplementation also led to improvement in their psoriasis. The diet categories that led to the most skin improvement included the Pagano, vegan, and paleolithic diets [15]. Another study aimed to compare dietary habits between psoriatic patients and healthy controls [16]. Compared to healthy controls, psoriatic participants consumed less olive oil, berries, fish, seafood, and nuts, while consuming more dairy products and sugary soft drinks. When comparing psoriatic participants against each other, the study found that those with greater PASI scores consumed red meat, instant noodles, or belly meat more frequently than those with lower PASI scores [16]. Additional studies have also found that the supplementation of antioxidant-rich plant compounds, including carotenoids and isoflavones, may also improve psoriasis severity [18,19].

To the best of our knowledge, there are no clinical studies directly studying the effects of a plant-based diet on psoriasis. Interestingly, one cross-sectional study observed the effects of a Mediterranean diet, which consists of olive oil, vegetables and fruits, seafood, and nuts, on psoriatic subjects [17]. This study found that a higher consumption of olive oil, vegetables, fruits, seafood, and nuts led to improvement in psoriasis while increased consumption of red or processed meats worsened psoriasis. Although this study did not strictly investigate a plant-based diet, it suggests that diets with higher compositions of plant-based foods may influence psoriatic disease severity beneficially. There have also been two case studies demonstrating improvements in psoriasis after adopting a plant-based diet. In the first case, a 40-year-old female with psoriatic arthritis adopted a whole-food plant-based diet and was able to stop taking methotrexate approximately six months later, remaining symptom-free since February 2018 [32]. In the second case, a 47-year-old male with severe plaque psoriasis achieved remission of his disease after a 13-day, medically supervised water-only fast followed by the adoption of a whole-food plant-based diet [33]. There was significant improvement in plaques, nail bed psoriasis, arthritis, and weight loss. This patient has since maintained the diet without any new lesions to report.

There are several possible mechanisms that explain why a plant-based diet may reduce disease severity, including the dietary patterns previously described. Several studies have demonstrated that dietary interventions resulting in weight loss and reductions in metabolic derangement improve psoriasis [34]. Thus, a plant-based diet, which has been shown to promote weight loss and reverse metabolic disease [35], would likely be beneficial for improving psoriasis. Eliminating animal-based products also greatly reduces the intake of saturated fatty acids, which have been shown to have inflammatory effects [36]. Many plant-based foods contain carotenoids and isoflavones that improve the skin’s antioxidant and anti-inflammatory activity. For example, soy is rich in isoflavones, such as genistein, and has been shown to support skin health in studies evaluating photoaging [37], dyspigmentation [38], and wound healing [39]. Sources of carotenoids include green leafy vegetables, colored fruits, and vegetables, which have also been studied for ameliorating deleterious effects of ultraviolet radiation such as sunburns and photoaging [40]. Increased consumption of fiber associated with a plant-based diet may also ameliorate gut-dysbiosis-related mechanisms driving systemic inflammation. Moreover, fermented plant-based foods may contain probiotics that target the gut–skin axis. The role of probiotic use in psoriasis is supported by numerous studies demonstrating that probiotic supplementation reduces psoriasis severity [8]. Plant-based foods may also lead to increased potassium intake, which may have anti-inflammatory properties [41].

### 3.2. Acne Vulgaris

Acne vulgaris is a very common skin condition affecting the pilosebaceous unit that is associated with a high cutaneous, psychological, and social burden of disease [42]. Despite being the most common diagnosis made by dermatologists, the pathogenesis and potential causes for acne are complex and remain under discussion [43]. One mechanism that remains a topic of considerable interest is the influence of diet on acne pathogenesis and severity.

In one 2023 cross-sectional study on diet and acne, researchers collected data on how often subjects with acne vulgaris and a non-acne control group consumed various foods and how they perceived the impact of those foods [20]. Subjects reported that probiotics, fruits, and vegetables had a positive effect on their acne, whereas fried food, chocolate, alcohol, and soft drinks posed a negative effect. Similar findings were seen in previous acne studies collecting self-reported data, including a 2001 study asking recent medical graduates which factors exacerbate their acne [44]. A total of 41% of students claimed that diet was an important factor, and 12% of those students specifically cited chocolate. A 2003 study compared subjects’ self-reported dietary quality with an independent evaluation of their acne; an inverse association was found between quality and acne exacerbation and severity [45].

To evaluate the relationship between diet and acne without relying on self-reported triggers, researchers from the 2023 study collected the consumption frequencies of food items across four weeks and calculated the cut-off frequencies and corresponding odds ratios for each item [20]. Exceeding the cut-off frequency for pork (≥4/week), beef (≥4/week), and cornflakes (≥1/month) increased the odds of acne, whereas exceeding the threshold for other food items including pasta (≤2/month), vegetables (≤3/week), coffee (≤5/week), and fruit (≤1/day) decreased the odds of acne by a statistically significant amount. The study also uncovered associations between diet and biological markers implicated in acne pathogenesis. Elevated levels of insulin-like growth factor 1 (IGF-1) were observed in acne patients, and IGF-1 levels were positively correlated with dairy intake. This suggests a link between dietary factors such as dairy consumption and the biological processes involved in acne development. Using the odds ratios, the study proposed an acne nutrition score for patients based on dietary habits. By quantifying the impact of diet on acne risk, clinicians can offer more tailored dietary recommendations to patients.

A 2005 case–control study examined the hypothesis that milk intake was associated with teenage acne, collecting data from nurses (n = 47,315) about their adolescent diets and whether they had been diagnosed with severe acne by a physician during adolescence. Multivariate models comparing the intake of whole, powdered, low-fat, and skim milk to the diagnosis of acne were adjusted for energy, body mass index, present age, and age of menarche. The results showed that higher total milk intake was associated with more severe acne, and acne prevalence decreased as the milk fat content decreased. Vitamin D supplementation and total energy intake were significantly positively associated with acne prevalence, and there were no associations between acne and soda, fries, pizza, or chocolate in this study. As a result, the authors hypothesized that hormones found in milk were responsible for the association with acne [46].

A 2008 prospective cohort study found an association between dairy intake and acne. The results showed a positive association between skim milk consumption and acne in teenage boys, with prevalence ratios ranging from 1.16 to 1.19 for various milk types. The findings suggest skim milk may contain hormonal constituents influencing endogenous hormones [47]. Together these data support the hypothesis that a plant-based diet may have an important impact on regulating the hormonal component of acne.

A cross-sectional study from 2002 found a lower acne prevalence in rural populations compared to Westernized society, studying the Kitavan islanders of Papua New Guinea and the Aché peoples of Paraguay. The islanders’ consumption of certain food items, including dairy, coffee, alcohol, sugar, salt, and oils, was far lower, and no cases of acne were seen in either population. Their diet primarily consisted of root vegetables, fruit, and fish. The Aché diet consisted primarily of manioc, maize, peanuts, and rice, with a smaller constituent consisting of flour, sugar, and meat. The authors suggested that low fat and sugar intake could explain the low acne prevalence across these populations [48].

### 3.3. Hidradenitis Suppurativa

Hidradenitis suppurativa (HS) is a chronic inflammatory disease hypothesized to be driven by inflammatory cytokines and hormonal changes. HS is associated with comorbidities including obesity, metabolic syndrome, dyslipidemia, and polycystic ovarian syndrome (PCOS). Due to the challenging nature of managing HS, the role of diet in HS has become a topic of interest.

Although the literature on plant-based diets and HS is still emerging, several studies have discussed common dietary patterns among HS patients, which may reveal common triggers. For example, one 2020 survey study sought to identify exacerbating and alleviating foods for HS subjects [21]. Exacerbating foods included sugary sweets, bread, pasta, rice, dairy, and high-fat foods. Alleviating foods included vegetables, fruits, and lean white meat [21].

Due to the association of obesity with HS severity, dietary interventions that promote weight loss have been shown to improve HS symptoms [7,49]. This is one mechanism by which a balanced plant-based diet may improve HS. Clinical trials and observational research have both demonstrated that plant-based diets promote weight loss and individuals consuming plant-based diets tend to have lower BMI than those that do not [50,51]. Another activating trigger for HS is thought to be related to androgen-induced obstruction of follicular ducts, which is worsened by hyperinsulinemia and IGF-1 [52]. Therefore, low glycemic diets and the avoidance of simple carbohydrates may benefit HS. Moreover, dairy consumption has been shown to activate IGF-1. These mechanisms align with the dietary patterns previously mentioned, which described the worsening of disease with the consumption of high glycemic foods and dairy. Notably, one study investigating a dairy-free diet in HS patients saw that the intervention led to an improvement in disease severity in 83% of subjects [25].

Interestingly, three studies investigated the role of a Mediterranean diet (MD) in HS disease activity. The MD is characterized by a high proportion of plant-based foods such as fruits, vegetables, legumes, bread, fruit, nuts, and extra virgin olive oil (EVOO) coupled with a lower consumption of meat, dairy, alcohol, and eggs [53]. An MD has also been shown to promote weight loss [54]. One 2019 cross-sectional study characterized the nutritional patterns of HS patients with low adherence to the MD, revealing an excess of simple carbohydrates and saturated fats, along with a lower intake of complex carbohydrates and monounsaturated and omega-3 polyunsaturated fats [22]. The degree of adherence to the MD was negatively associated with HS severity, as measured by HS Sartorius scores [22]. Another 2022 cross-sectional study demonstrated that higher adherence to an MD was associated with reduced HS disease activity, per self-reported Hurley and International Hidradenitis Suppurativa Severity Score System (IHS4) [23]. Notably, the use of EVOO as the main lipid to cook with also led to a significant reduction in self-reported disease activity [23]. EVOO contains monounsaturated fatty acids and bioactive phenolic compounds that have anti-inflammatory and antioxidant properties [55]. These mechanisms may explain the correlation of EVOO consumption with improved HS symptoms. These findings contrast a 2022 case–control study that did not correlate MD adherence with HS disease severity [24]. Nevertheless, these preliminary findings demonstrate a complex relationship between diet and HS. Proper adherence to the MD, which emphasizes the consumption of plant-based foods and less processed foods, may have a role in HS management.

There is also evidence suggesting that gut dysbiosis is associated with HS severity [56], and thus a diet that supports the gut microbiome may be beneficial in disease management. For many of the same reasons discussed in psoriasis and acne, a well-balanced plant-based diet containing fiber, antioxidants, flavonoids, and probiotics could reduce inflammation and comorbidities associated with HS. Nevertheless, more research is needed to determine how plant-based foods may influence HS.

### 3.4. Atopic Dermatitis

Atopic dermatitis (AD) is a common inflammatory skin condition often associated with other atopic diseases including asthma and food or seasonal allergies. In combination with genetic factors, AD in children is often theorized to be influenced by dietary exposures early in life. For example, the consumption of breast milk has been suggested to be protective against AD, likely due to the passing on of immunologic components from the mother [57]. The first therapeutic dietary modification is simply avoiding food allergens and known triggers. However, recent studies exemplify the utility of plant-based foods in managing AD.

The increased consumption of fast foods and red meat burgers has been associated with higher AD prevalence in children [26,27]. There are several reasons why a diet that includes regular fast food intake may worsen or trigger AD. Typically, regular fast food intake is associated with increased caloric intake which can lead to obesity. Excess body fat alone is a known sensitizer for allergens [58]. Additionally, increased consumption of fatty acids has been shown to upregulate inflammatory pathways [59]. High-fat, low-fiber diets, such as animal-based diets, are also known to promote gut dysbiosis which limits the production of beneficial short-chain fatty acids, further augmenting the inflammatory response. Given this and the role of the skin microbiome in AD severity, regulating the gut microbiome via dietary changes may be one method to target AD symptoms.

One study in 2000 investigated the effects of a low-energy diet (1085 calories) composed of fresh vegetable juice, brown rice porridge, kelp powder, soybean curd, sesame paste, and non-refined salt for 8 weeks in those with AD [28]. Interestingly, all the foods in this low-energy diet were plant-based, demonstrating that all nutritional needs can be met with a plant-based diet. Furthermore, participants experienced weight loss, reduced BMI, reduced systolic blood pressure, and significant reductions in the scoring atopic dermatitis (SCORAD) index [28]. This diet had several notable components including fiber, protein, fermented foods containing probiotics, and low glycemic carbohydrates. Implementing a diet rich in fiber and fermented plant-based foods may have beneficials effects on AD via these proposed mechanisms.

Fruits and vegetables are rich in fiber, antioxidants, flavonoids, and bioactive compounds that exert anti-inflammatory effects and improve gut dysbiosis. Moreover, probiotic supplementation has been shown to be therapeutic in patients with AD [60,61,62]. Thus, fermented foods rich in probiotics may act similarly to increase the production of short-chain fatty acids and influence functional pathways driving AD. There is a wide range of plant-based fermented foods including sauerkraut, kimchi, tempeh, natto, and miso, among others [63].

### 3.5. Skin and Wound Healing

The skin healing process involves several stages including an initial hemostasis stage, an inflammatory stage, a proliferative stage, and a final remodeling stage. There are several factors that can impair the healing process including systemic and chronic illness, stress, age, concurrent medications, drug use, and hormonal changes [64]. Studies evaluating the effect of nutrition and diet on skin healing are still emerging. However, one study in 2021 investigating the role of vegan versus omnivore diets on skin healing after photodynamic therapy (PDT) for actinic keratoses demonstrated that the vegan group had significantly higher local skin responses (characterized by edema, vesiculation, erythema, and desquamation) after 3, 7, and 30 days compared to the omnivore group [65]. These findings suggest that the vegan group is either more prone to inflammatory reactions or that their wound healing process is altered, possibly due to dietary differences. Another study from the same investigative group compared postsurgical skin healing in vegans and omnivores following surgical excision of nonmelanoma skin cancers [66]. After 6 months, the study found that vegans had a higher Scar Cosmesis Assessment and Rating (SCAR) score, more frequent wound diastasis, and more atrophic scares than omnivores, suggesting worse skin healing outcomes in vegans [66]. Both of these studies measured vitamin B12 and iron levels and noted that they were significantly lower in the vegan group. However, neither study measured or estimated protein intake, the quality of food intake, or the duration of the diet which could have influenced the results.

Another clinical study evaluated the results of facial fractional microneedle radiofrequency treatments among those who followed either a vegan or omnivore diet for at least 5 years [67]. The authors noted that the 25-OH vitamin D, vitamin B12, and ferritin levels were significantly lower in the vegan cohort and a vegan diet was associated with reduced reduction in the Fitzpatrick wrinkle scale and worsened ratings in the Patient’s Global Impression of Change. Notably, this study did not account for protein intake as this was not assessed or estimated among the participants.

In general, the studies evaluating the role of a plant-based diet in skin healing have poor accounting for protein intake which is important in protein and collagen synthesis. Food profiles were not assessed, and the intake of compounds such as carotenoids, polyphenols, fiber, and antioxidants may have influenced the outcomes. Interestingly, blood measurements of ferritin and vitamin B12 were significantly lower than omnivores in all three studies [65,66,67]. Serum iron and vitamin B12 are both involved in wound healing behavior and enzymatic activity regulation and thus a deficiency could contribute to healing time. Overall, it has been noted that there can be vitamin and nutrient deficiencies in vegan diets, including iron, vitamin B12, and lower protein intakes [68]. Future prospective studies should better account for protein intake. If a plant-based diet is adopted, patients should be counseled on adequate protein consumption and optimizing iron, vitamin B12, and 25-OH vitamin D levels in relation to skin and wound healing.

### 3.6. Nutritional Deficiencies

Among the nutritional deficiencies that can occur with a strict plant-based diet, the most reported are vitamin A, riboflavin, and vitamin B12 deficiency. Although deficiencies in these nutrients can have significant health outcomes, there are many plant-based sources or easily available supplements of these vitamins. Thus, with the proper education and adequate planning, concerns for nutritional deficiencies are drastically reduced.

Vitamin A is a fat-soluble compound intricately involved with cutaneous processes, including epithelial differentiation and immunomodulation [69]. Vitamin A deficiency may lead to epithelial keratinization, which manifests clinically as generalized xerosis, phrynoderma, and hair casts [70]. While a highly restrictive diet may be deficient in vitamin A, the Academy of Nutrition and Dietetics confirms that an appropriately planned vegan diet is nutritionally adequate for all stages of life [71]. Plant-based sources of vitamin A include tomatoes, bell peppers, leafy greens, and cantaloupe.

Riboflavin, also known as vitamin B2, is a water-soluble nutrient essential for various oxidation–reduction reactions [72]. Deficiency in riboflavin typically manifests with symptoms such as angular cheilitis, glossitis, and sometimes a reddish, scaly rash in the genital and thigh area, sparing the midline. In embryonic development, riboflavin deficiency can lead to abnormalities in muscles, bones, and the gastrointestinal tract. In adults, it may cause anemia, impaired iron absorption, nerve damage, and peripheral neuropathy [72]. While studies suggest lower riboflavin intake in vegan diets compared to non-vegan ones, clinical deficiency is rare in adults. However, a case study highlighted severe hypoglycemia and lactic acidosis in a newborn due to maternal riboflavin deficiency, emphasizing the importance of adequate intake during pregnancy, especially for vegan mothers [73]. Plant-based sources rich in riboflavin include asparagus, bananas, beans, broccoli, figs, kale, and lentils. Pregnant women following a vegan diet may require additional supplementation.

Vitamin B12, otherwise known as cobalamin, is a water-soluble vitamin with important roles in the nervous system, hematological system, and skin [74]. Cutaneous manifestations of vitamin B12 deficiency include pigmentary changes, hair/nail changes, and glossitis. Many dermatoses, including vitiligo, acne, and AD, are related to altered vitamin B12 levels [74]. High dose supplementation of vitamin B12 has also been linked to acne, rosacea, and allergic reactions [74]. Although most dietary sources for vitamin B12 come from animal products, some dried green and purple nori, nutritional yeast, fortified cereals, plant milks, and tempeh contain vitamin B12 [75]. Measuring B12 levels may be necessary to determine if additional supplementation is needed.

### 3.7. Omega-3 and Omega-6 Fatty Acids

**Figure 3:**
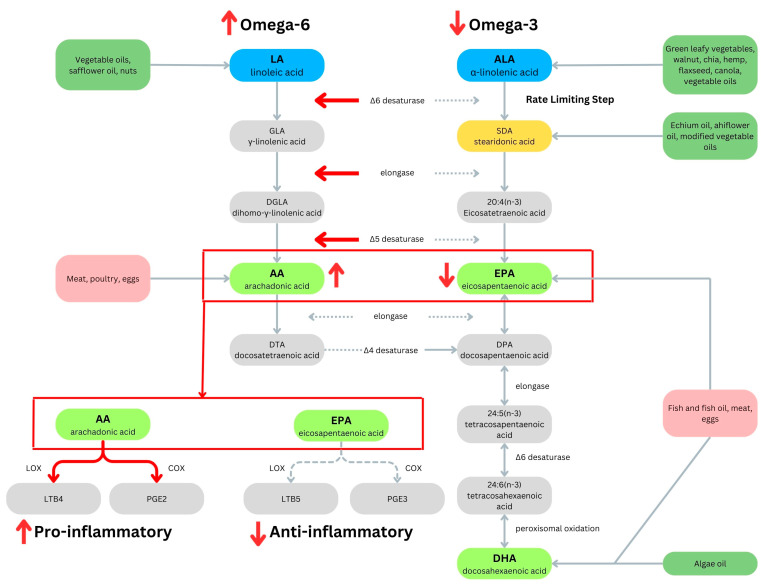
Omega-6 and omega-3 pathways demonstrating shared enzymes and food sources of LA, ALA, AA, EPA, and DHA. When the ratio of omega-6 to omega-3 is high (>4:1), enzymes are used up in the LA pathway, which results in a proinflammatory state. This figure was created on Canva (Sydney, Australia).

Omega-3 and -6 fatty acids are essential fatty acids that must be consumed in the diet due to the lack of enzymes in mammals for omega-3 desaturation [76]. Omega-6 fatty acids can both promote and reduce inflammation, depending on the specific type and context of their metabolism [77]. However, eicosanoid products derived from omega-6 fatty acids tend to be more potent mediators of thrombosis and inflammation than those derived from omega-3 fatty acids, thereby resulting in a prothrombotic and proinflammatory profile when omega-6 fatty acids dominate (Figure 3) [78]. Both play key roles in modulating adipogenesis, inflammation, insulin resistance, and cannabinoid production. A ratio of approximately 4:1 in omega-6 to omega-3 fatty acids is optimal for reducing inflammation, allergies, and autoimmune reactions [79].

Multiple studies have evaluated the effect of shifting omega-3 and omega-6 polyunsaturated fatty acids through dietary modifications, especially in cutaneous physiology and disease. For example, omega-3 supplementation in combination with conventional treatment led to improvements in PASI scores, pruritus, and Dermatological Life Quality Index (DLQI) scores in psoriatic patients [80,81]. Moreover, an increase in the ratio of omega-6 to omega-3 fatty acids to the 20:1 ratio that is present in a current Western diet significantly increases the risk for obesity, a known trigger for psoriasis, AD, and HS [78].

Plant-based sources of omega-3 fatty acids are primarily represented by alpha-linolenic acid (ALA) which is found in green leafy vegetables, flax seeds, chia seeds, perilla, and walnuts [82]. ALA is metabolized to eicosapentaenoic acid (EPA) and docosahexaenoic acid (DHA), which are found in the oils of fatty fish [78]. The conversion of ALA in plant sources to EPA and DHA is limited via the rate-limiting step of converting ALA to stearidonic acid (SDA). Thus, omega-3 fatty acids are often supplemented utilizing fish oil. In order to overcome this, a plant-based approach to supplementing omega-3 fatty acids might consider a few modifications. For example, one could increase the intake of ALA-rich plant foods such as flaxseeds, chia seeds, and walnuts. There are also foods and supplements that are enriched in SDA, such as ahiflower and echium oil [83,84]. Ahiflower and echium oil are derived from the seeds of the *Buglossoides arvensis* and *Echium platagineum* plants, respectively [83,85]. These bypass the rate-limiting ALA to SDA step and increase EPA and DHA levels more effectively. Furthermore, there are plant-based sources of DHA, which are derived from algae and can also augment EPA levels [86]. Increasing DHA levels augment EPA levels by a process called retroconversion, which is the direct conversion of DHA to EPA via peroxisomal β-oxidation [87].

### 3.8. Protein Intake

Protein is a highly versatile macro-nutrient that serves a broad range of physiological functions including structural components, enzymes, hormones, and transporters. Enzymes regulate metabolic pathways by catalyzing various biochemical reactions. Hormones allow tissues to communicate and maintain homeostasis. A severe deficiency in total protein leads to Kwashiorkor syndrome, which is a condition seen most often in pediatric populations within developing countries and is rarely observed in developed countries such as the United States [88]. The dermatological effects of Kwashiorkor include skin lesions near points of friction characterized by darkly pigmented patches also known as “flaky paint” dermatosis [89].

While animal-based protein is considered “complete” due to the presence of all essential amino acids, each amino acid can be obtained with an entirely plant-based diet by consuming multiple sources of protein over the course of a day or days. Table 2 shows some of the common plant-based protein sources, as well as their protein contents per 100 g [90].

## 4. Conclusions

In summation, the literature supports several mechanisms by which a well-balanced plant-based diet may improve inflammatory skin conditions and their associated comorbidities. Specifically, plant-based foods may support the gut microbiome, exert antioxidant effects, reduce glycemic load, and promote weight loss, all of which downregulate systemic inflammation driving psoriasis, acne, HS, and AD. Moreover, a plant-based diet with appropriate supplementation can address concerns of nutritional deficiencies. Therefore, a plant-based diet may be one component of a therapeutic regimen for inflammatory skin conditions. Nevertheless, further clinical research is needed to expand the evidence for plant-based diets in dermatology. Research with analysis relating back to the gut microbiome and metabolome would reveal additional mechanisms that may explain connections between diet and the skin.

## Figures and Tables

**Table 1:** Summary of exacerbating and alleviating dietary factors.

Skin Condition	Exacerbating	Alleviating
Psoriasis	- Alcohol, gluten, and nightshade vegetables [15] - Dairy products and sugary soft drinks [16] - Red meat, instant noodles, and belly meat [16]	- Pagano, vegan, paleolithic diets, and omega-3 fatty acids [15] - Mediterranean diet [17] - Foods rich in carotenoids and flavonoids [18,19]
Acne Vulgaris	- Fried foods, chocolate, alcohol, soft drinks [20] - Pork, beef, and cornflakes [20] - Dairy products [20]	- Probiotics, fruits, and vegetables [20] - Pasta, vegetables, coffee, and fruit [20]
Hidradenitis Suppurativa	- Sugary sweets, bread, pasta, rice, dairy, and high-fat foods [21] - Meat, dairy, alcohol, and eggs [22,23,24]	- Vegetables, fruits, and lean white meat [21] - Low glycemic, no-dairy diet [25] - Fruits, vegetables, legumes, bread, fruit, nuts, and extra virgin olive oil [22,23,24]
Atopic Dermatitis	- Fast food and red meat burgers [26,27]	- Low-energy plant-based diet [28]

**Table 2:** Common plant-based foods and protein content [90].

Food	Protein Content per 100 g
Soybeans	18.2 g
Firm tofu	10.9 g
Black beans	8.86 g
Lentils	9.02 g
Chickpeas	8.86 g
Quinoa	4.4 g
Almonds	21.2 g
Oats (non-fortified)	13.2 g
Raw spinach	2.86 g
Raw spirulina	5.92 g

## Data Availability

No new data were generated for this review, but studies were reviewed from publicly available databases.
